# Characterizing distinct profiles of immune and inflammatory response with age to Omicron infection

**DOI:** 10.3389/fimmu.2023.1189482

**Published:** 2023-06-30

**Authors:** Lina Zhang, Zhanwen Wang, Feng Lyu, Chun Liu, Chunhui Li, Wei Liu, Xinhua Ma, Jieyu Zhou, Xinyu Qian, Zhaoxin Qian, Yong Lu

**Affiliations:** ^1^ Department of Critical Care Medicine, Xiangya Hospital, Central South University, Changsha, Hunan, China; ^2^ National Clinical Research Center for Geriatric Disorders, Xiangya Hospital, Central South University, Changsha, Hunan, China; ^3^ Hunan Provincial Clinical Research Center for Critical Care Medicine, Xiangya Hospital, Central South University, Changsha, Hunan, China; ^4^ School of Computer Science and Engineering, Central South University, Changsha, Hunan, China; ^5^ Respiratory and Critical Care Medicine Department, The Third Xiangya Hospital of Central South University, Changsha, Hunan, China; ^6^ Xiangya Hospital, Central South University, Changsha, Hunan, China; ^7^ Department of Radiology, Ruijin Hospital Luwan Branch, School of Medicine, Shanghai Jiaotong University, Shanghai, China

**Keywords:** COVID-19, omicron, immunology, inflammation, age

## Abstract

**Background:**

Understanding inflammatory and immune responses to Omicron infection based on age is crucial when addressing this global health threat. However, the lacking of comprehensive elucidation hinders the development of distinct treatments tailored to different age populations.

**Methods:**

1299 cases of Omicron infection in Shanghai were enrolled between April 10, 2022 and June 3, 2022, dividing into three groups by ages: Adult group (18-59 years), Old group (60-79 years), and Elder group (≥ 80 years). Laboratory data including inflammatory cytokines, cellular, and humoral immunity were collected and analyzed.

**Results:**

The mean age of Adult, Old, and Elder groups were 44.14, 69.98, and 89.35 years, respectively, with 40.9% being men. The Elder group patients exhibited higher white blood cell (WBC) counts and elevated levels of inflammatory cytokines, but their lymphocyte counts were relatively lower. In comparison to the Old group patients, the Elder group patients demonstrated significantly lower CD3^+^ T-cell counts, CD3^+^ T-cell proportion, CD4^+^ T-cell counts, CD8^+^ T-cell counts, and CD19^+^ B-cell counts, while the NK-cell counts were higher. Omicron negative patients displayed a higher proportion of CD19^+^ B-cells and higher levels of Complement-3 and IL-17 compared to the positive patients in the Old group. Omicron negative patients had lower WBC counts, CD3^+^CD8^+^ T-cells proportion, and the levels of serum amyloid A and IgA in the Elder group, but the CD4^+^/CD8^+^ ratio was higher.

**Conclusions:**

Our study identified the distinct profiles of inflammatory and immune responses to Omicron infection varying with age and highlighted the diverse correlations between the levels of various biomarkers and Omicron infected/convalescent patients.

## 1 Introduction

The global coronavirus (COVID-19) pandemic, caused by SARS-CoV-2, has prompted extensive research into characteristics immunological and inflammatory associated with SARS-CoV-2 infection (1–5). Lymphopenia has been observed in the majority of COVID-19 patients, and higher plasma levels of pro-inflammatory cytokines has been detected in severe infection patients (1, 6–8). The adaptive immune system, particularly CD4^+^ T cells and CD8^+^ T cells, has been proved to play a decisive role in regulating COVID-19 severity (9–12). Omicron (B.1.1.529), the latest SARS-CoV-2 variant of concern (VOC) displaced the most prevalent VOC, Delta (B.1.617.2) at the end of 2021 (13, 14). With over 30 coding mutations in its spike proteins, Omicron exhibits greater capacity of transmission and reinfection compared to previous VOCs (15, 16). Previous studies have indicated that the broad and magnitude of Omicron cross-reactive T cells had a similar profile for Beta (B.1.351) and Delta (B.1.617.2) variants (9, 17, 18). Both the humoral and cellular immune responses are involved in COVID-19 infected individuals (2). Additionally, it has been observed that Omicron infected patients tend to be older and have a higher prevalence of immunocompromised conditions compared to those infected with Delta (19). Shin J et al. found that disease severity and mortality in younger COVID-19 patients (age < 65) and older patients (age > 65) were associated with discrete immune mechanisms (8). However, the extent to which Omicron infection elicits different immunological and inflammatory profiles in various age populations, especially in individuals older than 60 years, has not been fully elucidated. Moreover, the correlations between the immune cells and inflammatory cytokines in the Omicron infection and convalescent individuals remain unclear. Further investigation is required to comprehensively understand these aspects.

In this study, we primary objective was to evaluate the immunological and inflammatory biomarkers during hospitalization to characterize the immune and inflammation responses to Omicron infection across different age populations. To provide a more comprehensive understanding of the responses to Omicron infection in the population over 60 years old, we introduced novel immunological and inflammatory markers specifically. Furthermore, the correlations between the immune cells and inflammatory cytokines in the Omicron infection individuals and convalescent individuals were explored.

## 2 Materials and methods

The Ruijin Hospital Luwan Branch Ethics Committee of Shanghai JiaoTong University Schoo1 of Medicine approved this study. Because COVID-19 is a notifiable disease, individual patient consent was waived.

### 2.1 Study subjects

This study was conducted in Ruijin Hospital Luwan Branch, School of Medicine, Shanghai Jiaotong University, and involved the enrollment of hospitalized patients diagnosed with Omicron between April 10, 2022, and June 3, 2022. Reverse transcription-polymerase chain reaction (RT–PCR) was used to detect the *ORF* and *N* gene of Omicron. The cycle threshold (Ct) values obtained from the RT-PCT assays were recorded. A confirmed case of Omicron infection was defined by a positive result of PT-PCR assay (i.e., Ct value < 35 for both *ORF* and *N* gene) of a specimen collected on a nasal or throat swab specimens (13). Demographic and laboratory data were collected from electronic medical records. In order to characterize the immune cells and inflammatory cytokines response to Omicron infection in different ages, especially for the elder people, the enrolled patients were divided into three groups: Adult group (18-59 years old), Old group (60-79 years old), and Elder group (≥ 80 years old).

To establish a baseline for comparison with the immune response elicited by Omicron infection, we retrieved laboratory results of the uninfected population from 2007 April to 2021 January in the Health Management Center of Xiangya Hospital, Central South University.

### 2.2 Sample preparation and examination

Blood for functional immune and inflammation studies were obtained within 2 weeks of hospitalization and discharge. The peripheral blood specimens were prepared for the blood routine, plasma cytokines, humoral and cellular immunity testing, respectively. For the assessment of inflammatory cytokines, the following markers were measured: C-reactive protein (CRP), serum amyloid A (SAA), procalcitonin (PCT), interleukin-1β (IL-1β), interleukin-2 (IL-2), interleukin-4 (IL-4), interleukin-5 (IL-5), interleukin-6 (IL-6), interleukin-8 (IL-8), interleukin-10 (IL-10), interleukin-12p70 (IL-12p70), interleukin-17 (IL-17), interferon-α (INF-α), interferon-γ (INF-γ), and tumor necrosis factor-α (TNF-α). Antibodies and main complements included IgA, IgM, IgG, complement C3 (C3), and complement C4 (C4), which reflected the humoral immune response. ELISA assays were utilized to measure the levels of inflammatory cytokines, antibodies, and complements in accordance with manufacturer’s instruction. For the cellular immunity detection, T-lymphocytes (CD3^+^ T) with its subpopulations (CD4^+^ T, CD8^+^ T, CD3^+^CD4^+^ T, CD3^+^CD8^+^ T), B-lymphocyte (CD19^+^ B) and NK cell number were measured by flow cytometry. The proportion of CD3^+^ T, CD3^+^CD4^+^ T, CD3^+^CD8^+^ T, CD19^+^ B, and NK, as well as the ratio of CD4^+/^CD8^+^ T, were calculated respectively.

*ORF* and *N* genes of each patient were examined by RT-PCR every 24 hours. An Omicron positive patient was considered as negative or convalescent when the Ct values of both *ORF* and *N* genes are larger than 35 for two consecutive tests (test interval > 24 hours).

### 2.3 Statistical analysis

GraphPad Prism (version 8.0.1) was utilized for statistical analysis. Continuous data were reported as medians, or mean ± standard deviation (SD) depending on the data distribution. Categorical variables were summarized as counts. Prior to performing comparisons, the normality of data was assessed. For normally distributed data, changes in the levels of biomarkers between two groups were analyzed using an unpaired *t* test. For data that did not follow a normal distribution, the nonparametric Mann-Whitney test was employed. Comparisons among three or more groups were analyzed using ANOVA or Kruskal-Wallis test according to the data normality. The relation of the inflammatory and immune markers with clinical outcomes was analyzed using generalize linear model (Stata/MP 14.0, TX, USA). P < 0.05 was considered statistically significant. To better describe the characteristics of biomarkers change during hospitalization, the cumulative distribution function (CDF) was plotted with Matplotlib, allowing for a better understanding of the characteristics and trends in biomarker levels over time.

## 3 Results

### 3.1 Characteristics and laboratory finding of the uninfected population and the Omicron cohorts at the admission

**Figure 1:**
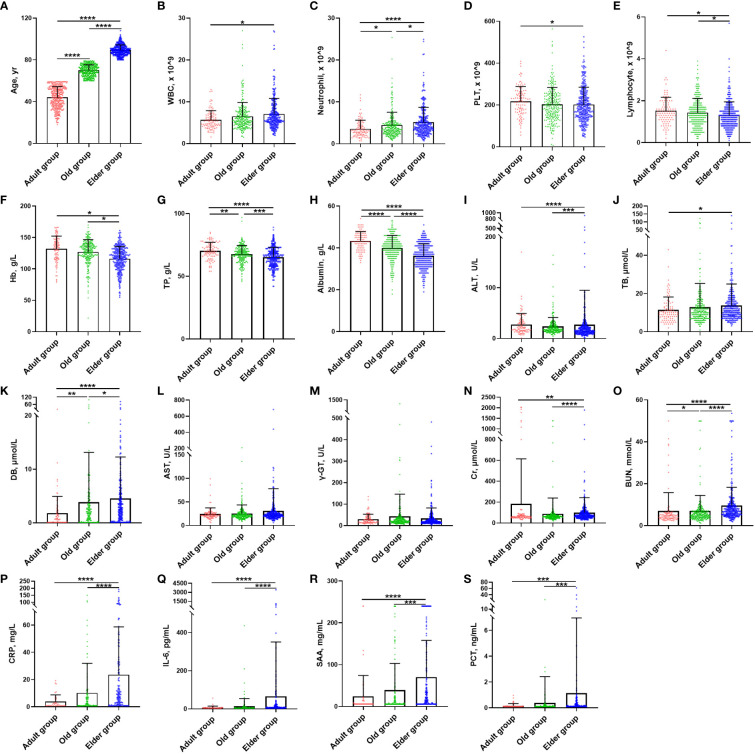
Demographic characteristics and laboratory findings of Omicron infection patients at study entry. **(A)** The mean age of the Adult, Old, and Elder groups were 44.14 ± 10.26 years (n = 382), 69.98 ± 5.34 years (n = 445), and 89.35 ± 4.93 years (n = 472). **(B)** The Elder group had higher WBC counts than the Adult group. **(C)** The Elder group had the highest neutrophils count compared with the Adult and Old groups. **(D)** The Adult group had higher platelet counts compared with the Elder group. **(E)** The lymphocyte count for the Elder group were lower than the Adult and Old groups. **(F)** The levels of hemoglobin for the Elder group were lower than the Adult and Old groups. **(G)** The levels of total protein for the Elder group were lower than the Adult and Old groups. **(H)** The levels of albumin for the Elder group were lower than the Adult and Old groups. **(I)** The Adult group had higher levels of ALT than the Elder group, but the Old group had lower levels of ALT than the Elder group. **(J)** The Elder group had higher levels of total bilirubin than the Adult group **(K)** The Elder group had the highest levels of direct bilirubin compared with the Adult and Old group. **(L)** There were no significant differences in the AST levels among the three groups. **(M)** There were no significant differences in the γ-glutamyltransferase levels among the three groups. **(N)** The Adult group had higher levels of serum creatinine than the Elder group, but the Old group had lower levels of ALT than the Elder group. **(O)** The levels of blood urea nitrogen was the highest in the Elder group. **(P)** CRP levels were higher in the Elder group than the Adult and Old group. **(Q)** IL-6 levels were higher in the Elder group than the Adult and Old group. **(R)** SAA levels were higher in the Elder group than the Adult and Old group. **(S)** PCT levels were higher in the Elder group than the Adult and Old group. In figure **(B–O)**, 99 individuals were tested in the Adult group, 251 individuals in the Old group, and 308 individuals in the Elder group. In figure **(P–S)**, 54 individuals were tested in the Adult group, 156 individuals in the Old group, and 186 individuals in the Elder group. Data were shown as mean ± SD. *P < 0.05, **P < 0.01, ***P < 0.001, and ****P < 0.0001; WBC, white blood cell; PLT, platelet; Hb, hemoglobin; TP, total protein; ALT, Alanine aminotransferase; AST, Aspartate aminotransferase; TB, total bilirubin; DB, direct bilirubin; γ-GT, γ-Glutamyltransferase; Cr, serum creatinine; BUN, blood urea nitrogen; CRP, C-reactive protein; IL-6, interleukin-6; SAA, serum amyloid A; PCT, procalcitonin.

A total of 1299 cases diagnosed with Omicron infection were included in this study. Among them, there were 382 cases in the Adult group, 445 cases in the Old group, and 472 cases in the Elder group (**Supplementary Table 1**). The mean ( ± SD) age of overall Omicron cohort was 68.34 ± 20.86 years, 44.14 ± 10.26 years for Adult group, 69.98 ± 5.34 years for Old group, and 89.35 ± 4.93 years for Elder group (**Figure 1A**). Among the participants, 40.9% were men. In the uninfected control population, a total of 6873 cases were enrolled, with the mean age of 67.38 ± 18.45 years. Specifically, the mean age was 44.0 ± 9.74 years for the Adult group, 67.75 ± 5.25 years for the Old group, and 85.33 ± 5.27 years for the Elder group (**Supplementary Table 2**). Comparing the uninfected population with Omicron-infected patients, total white blood cell (WBC) counts and neutrophil cell counts were similar in the uninfected population (**Supplementary Table 2**), which increased with age after Omicron infection (**Figures 1B, C**, **Supplementary Table 1**). The Omicron patients in the Elder group had lower levels of platelets compared to those in the Adult group (**Figure 1D**), which was in contrast to the fundings in the uninfected population (**Supplementary Table 2**). Additionally, the total lymphocyte counts, hemoglobin (Hb), total protein, and albumin declined compared to the uninfected population, with the lowest levels in the Elder group (**Figures 1E–H**). The Adult group patients had higher levels of alanine aminotransferase compared to the Elder group (**Figure 1I**), while the levels of total bilirubin and direct bilirubin were lower than those in the Elder group (**Figures 1J, K**). There were no significant differences in the levels of aspartate aminotransferase and γ-glutamyltransferase among the three groups (**Figures 1L, M**). The Adult group showed relatively higher serum creatinine levels compared with that of the Elder group, while the blood urea nitrogen level was the highest in the Elder group (**Figures 1N, O**). Furthermore, the Elder group had higher levels of circulating pro-inflammatory cytokines, including CRP, IL-6, SAA, and PCT when compared with that of the Adult and Old groups (**Figures 1P–S**).

**Figure 2:**
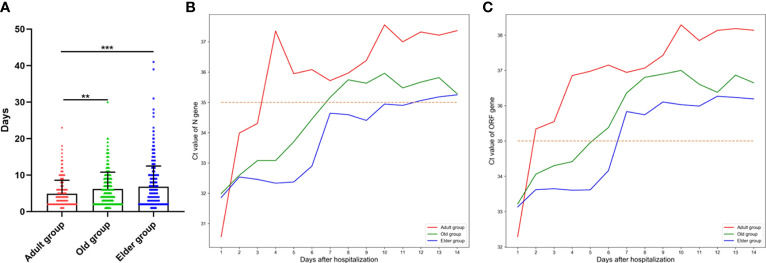
**(A)** The days taken for the Omicron infection patients turning negative for the Adult group (4.91 ± 3.67 days, n = 212) was significantly shorter than the Old and Elder groups (6.16 ± 4.63 days, n = 376; 6.8 ± 5.70 days, n = 415). **(B)** The change of the mean Ct value of *ORF* gene in different age groups during hospitalization. **(C)** The change of the mean Ct value of *N* gene in different age groups during hospitalization. The mean Ct value of *ORF* and *N* genes exhibited a rapid change after hospitalization in the Adult group, whereas the change of Ct value was much slower in the Old and Elder group. Data were shown as mean ± SD. **P < 0.01 and ***P < 0.001. Bule line represents Adult group, green line represents Old group, and Orange line represents Elder group. Red dot line represents Ct value of 35.

The days taken for the Omicron infection patients in the Adult group to turn negative was significantly shorter than those in the Old and Elder groups (**Figure 2A**). Moreover, we analyzed the mean Ct value change of different ages during hospitalization (**Figures 2B, C**). The Ct value of *ORF* and *N* genes varied rapidly within 5 days after hospitalization in the Adult group, while the change of Ct value was much slower in the Old group. In the Elder group, a pronounced change of the Ct value was observed around day 5 after hospitalization. Moreover, the Adult group had lower initial Ct values of N and ORF genes compared to the Old and Elder groups.

### 3.2 The characteristic change for white blood cell, lymphocyte, and inflammatory cytokines in different age during hospitalization

We plotted the cumulative distribution function (CDF) curves of immunological and inflammatory at various time points (Day 1, Day 5, and Day 7 after hospitalization as well as discharge) in different ages group (**Supplementary Figure 1**). The CDF curve of WBC count for the Adult group located in the upper left quadrant compare to the curves for the Old and Elder groups at each timepoint, which indicated the WBC count of the Adult group was lower than that in the other two groups. Conversely, the CDF curve of lymphocyte count for the Adult group was located in the lower right quadrant compared to the curves for the Old and Elder groups at each timepoints, indicating the lymphocyte count of the Adult group was higher than that in the other two groups. As the inflammatory cytokines were not regularly measured for the Adult group, the CDF curves for this group were not as smooth as those for the Old and Elder groups. However, it is difficult to clearly distinguish the CDF curves of Old and Elder groups when assessing these laboratory biomarkers.

**Figure 3:**
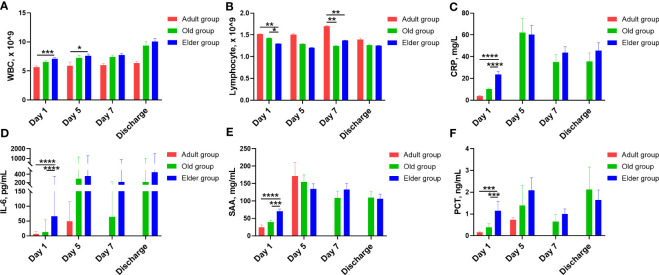
The characteristic change for white blood cell, lymphocyte, and inflammatory cytokines in different age during hospitalization. **(A)** Compared with the Elder group, total WBC counts were lower in the Adult group at Day 1 and Day 5 after hospitalization. **(B)** The total lymphocyte counts of the Adult and Old groups were higher than the Elder group at Day 1. At Day 7, the lymphocyte counts were higher in the Adult group than the Old and Elder groups. The temporal change patterns varied for the inflammatory cytokines levels. **(C)** CPR levels were higher in the Elder group than the Adult and Old group at Day 1. **(D)** IL-6 levels were higher in the Elder group than the Adult and Old group at Day 1. **(E)** SAA levels were higher in the Elder group than the Adult and Old group at Day 1. **(F)** PCT levels were higher in the Elder group than the Adult and Old group at Day 1. In figure **(A, B)**, 99 individuals were tested in the Adult group, 251 individuals in the Old group, and 308 individuals in the Elder group at day 1; 26 individuals were tested in the Adult group, 85 individuals in the Old group, and 149 individuals in the Elder group at day 5; 27 individuals were tested in the Adult group, 80 individuals in the Old group, and 188 individuals in the Elder group at day 7; 11 individuals were tested in the Adult group, 54 individuals in the Old group, and 115 individuals in the Elder group at discharge. In figure **(C–F)**, 54 individuals were tested in the Adult group, 156 individuals in the Old group, and 186 individuals in the Elder group at day 1; 4 individuals were tested in the Adult group, 22 individuals in the Old group, and 39 individuals in the Elder group at day 5; 23 individuals were tested in the Old group and 35 individuals in the Elder group at day 7; 26 individuals were tested in the Old group and 48 individuals in the Elder group at discharge. Data were shown as mean ± SE. *P < 0.05, **P < 0.01, ***P < 0.001, and ****P < 0.0001; WBC, white blood cell; CRP, C-reactive protein; IL-6, interleukin-6; SAA, serum amyloid A; PCT, procalcitonin.

To better compare the detailed changes among these different age groups, quantification analysis was also performed (**Figure 3**, **Supplementary Table 3**). We found that total WBC counts were lower in the Adult group compared to the Elder group at Day 1 and Day 5 after hospitalization (**Figure 3A**). Moreover, the WBC levels remained relatively stable for the Adult group during hospitalization, while they continued to increase during hospitalization in both the Old and Elder groups. In contrast, the total lymphocyte counts in the Adult and Old groups were higher than that in the Elder group at Day 1 (**Figure 3B**). By Day 7, the lymphocyte counts in the Adult group were higher than those in the Old and Elder groups, but they returned to the similar levels at discharge (**Figure 3B**). The temporal change patterns of the inflammatory cytokines levels varied among the different age groups. At Day 1, the levels of pro-inflammatory cytokines were the highest in the Elder group (**Figures 3C–F**). Although differences in the levels of CRP, IL-6, SAA, and PCT were observed among different age groups at Day 5, 7, and discharge, statistical significance was not reached. It is important to note that in the Adult group, there were fewer than three patients tested with the inflammatory cytokines at Day 7 and discharge, so the levels of the pro-inflammatory cytokines were not included in the analysis.

### 3.3 The characteristic change for novel immune and inflammatory markers in different age during hospitalization

**Figure 4:**
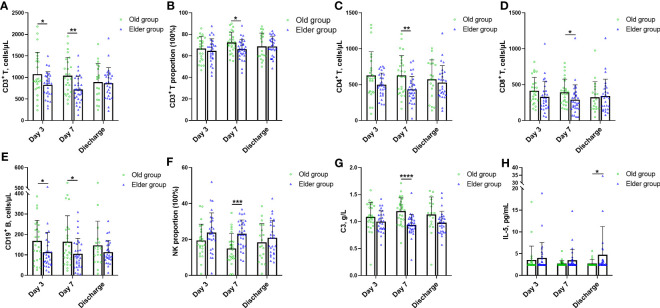
The characteristic change for novel immune and inflammatory markers in different age during hospitalization. **(A)** The Old group had higher CD3^+^ T counts than the Elder group at Day 3 and Day 7. **(B)** The proportion of CD3^+^ T in the Old group was higher than the Elder group at Day 7. **(C)** The counts of CD4^+^ T in the Old group were higher than the Elder group at Day 7. **(D)** The CD8^+^ T counts in the Old group were higher than the Elder group at Day 7. **(E)** The Old group had higher CD^+^ 19 B cell counts than the Elder group at day 3 and Day 7. **(F)** The NK proportion was lower in the Old group compared with the Elder group at Day 7. **(G)** The C3 levels were higher in the Old group than the Elder group at Day 7. **(H)** IL-5 levels in the Old group were higher than the Elder group at discharge. 24 individuals were tested in the Old group and 28 individuals in the Elder group at day 3; 26 individuals were tested in the Old group and 31 individuals in the Elder group at day 7; 19 individuals were tested in the Old group and 27 individuals in the Elder group at discharge. Data were shown as mean ± SD. *P < 0.05, **P < 0.01, ***P < 0.001, and ****P < 0.0001. NK, nature kill cell; C3, complement 3; IL-5, interleukin 5.

As mentioned previously, the CDF curves of the Old and Elder groups intersected when individually analyzing the WBC, lymphocyte, and inflammatory cytokines such as CRP, IL-6, SAA, and PCT, at the same time point during hospitalization. The detailed data further revealed that the Old and Elder groups had the similar levels of WBC, lymphocyte, and inflammatory cytokines. To explore whether there were differences in the immunological and inflammatory response of the Old and Elder groups, we introduced other biomarkers, including the cellular and humoral immunological and inflammatory markers (**Figure 4**, **Supplementary Table 4**). Initially, we focused on the cellular immune response to Omicron and found variations between the Old and Elder groups. The Old group had higher CD3^+^ T counts compared to the Elder group on both Day 3 and Day 7 (**Figure 4A**), but the proportion of CD3^+^ T in the Old group was higher than in the Elder group only at Day 7 (**Figure 4B**). In the aspect of the T cell subpopulation analysis, we observed that both CD4^+^ T and CD8^+^ T counts were higher in the Old group than in the Elder group at Day 7 (**Figures 4C, D**). Similar pattern was observed for the CD^+^ 19 B cells. The Old group had higher CD^+^ 19 B cell counts than in the Elder group at day 3 and Day 7 (**Figure 4E**). However, the NK proportion was lower in the Old group compared to the Elder group at Day 7 (**Figure 4F**). Among the biomarkers representing humoral immune response, we found a significant difference in C3 levels between the Old group and Elder group at Day 7 (**Figure 4G**). The levels of majority of the circulating anti-inflammatory and pro-inflammatory cytokines were similar in the Old and Elder group during hospitalization, except the IL-5 level at discharge (**Figure 4H**).

### 3.4 The correlation between Omicron infected/convalescent patients and immunological and inflammatory cytokines in different age population

**Figure 5:**
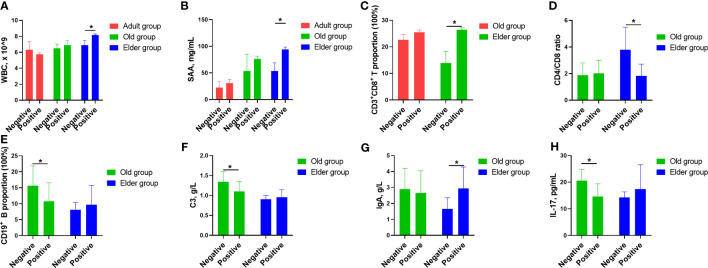
The correlation between Omicron infected/convalescent patients and immunological and inflammatory cytokines in different age population. **(A)** In the Elder group, the WBC counts in the positive patients were higher compared with the negative patients. **(B)** SAA levels in the positive patients were higher compared with the negative patients in the Elder group. **(C)** The positive patients in the Elder group had higher CD3^+^CD8^+^ proportion than the negative patients. **(D)** The ratio of CD4^+^/CD8^+^ was lower in the positive patients of the Elder group than the the negative patients. **(E)** The CD19^+^ proportion in the negative patients of the Old group was higher than the the positive patients. **(F)** The negative patients had higher levels of C3 compared with the positive patients in the Old group. **(G)** The IgA levels in the negative patients of the Elder group was lower than the positive patients. **(H)** IL-17 levels in the negative patients of the Old group was higher than the positive patients. Data were shown as mean ± SE. *P < 0.05. WBC, white blood cell; SAA, serum amyloid A; C3, complement 3; IL-17, interleukin 17.

We initially analyzed the markers which are routinely used in clinical practice, including WBC, lymphocyte, CRP, IL-6, SAA, and PCT (**Figure 5**, **Supplementary Table 5**). In the Elder group, the WBC and SAA levels in the positive patients were higher compared to negative patients (**Figures 5A, B**). Turing our attention to T cell and its subpopulation, the positive patients in the Elder group displayed a higher CD3^+^CD8^+^ proportion than negative patients (**Figure 5C**), while the ratio of CD4^+^/CD8^+^ was lower in the positive patients (**Figure 5D**). The CD19^+^ proportion in the negative patients of the Old group was higher than that in the positive patients (**Figure 5E**). Among the markers representing humoral immunity, the negative patients in the Old group had higher levels of C3 compared to the positive patients (**Figure 5F**). The IgA levels in the negative patients in the Elder group was lower than the positive patients (**Figure 5G**). Regarding the novel biomarkers, the negative patients in the Old group displayed higher levels of IL-17 than positive patients (**Figure 5H**).

### 3.5 Clinical outcomes and possible factor contributing to clinical outcomes

The mortality of the Adult group was 0.52%, 2.48% for the Old group, and 11.01% for the Elder group. The overall mortality was 5.0%. Considering different treatment would affect the tested parameters after hospitalization, clinical outcome, and faster recovery in the Adult group, we primarily analyzed the relation of the parameters tested by the 1st day after hospitalization with clinical outcomes. In the univariate analysis, factors that contributed to mortality positively included age, white blood cell, neutrophil, alanine aminotransferase, aspartate aminotransferase, total bilirubin, direct bilirubin, C-reactive protein, serum amyloid A, and procalcitonin. On the contrary, lymphocyte, hemoglobin, total protein, and albumin were negatively associated with mortality (**Table 1**). In the multivariate analysis, aspartate aminotransferase was found to positively relate to mortality.

**Table 1:** The relation of inflammatory and immune markers with clinical outcomes.

Variables	Univariate analysis			Multivariate analysis		
	Coeff	SE	p value	Coeff	SE	p value
Age	0.038	0.011	0.001			
WBC	0.014	0.002	< 0.001			
Neutrophil	0.015	0.002	< 0.001			
Lymphocytes	-0.017	0.008	0.029			
Hemoglobin	-0.001	0.0004	0.009			
Total protein	-0.003	0.001	0.024			
Albumin	-0.0069	0.001	< 0.001			
ALT	0.00076	0.0002	< 0.001			
AST	0.0016	0.0003	< 0.001	0.0058	0.001	< 0.001
TB	0.004	0.0008	< 0.001			
DB	0.0077	0.001	< 0.001			
CRP	0.001	0.0004	0.002			
IL-6	0.0003	0.00004	< 0.001			
SAA	0.00038	0.0001	0.001			
PCT	0.007	0.003	0.031			

WBC, white blood cell; ALT, alanine aminotransferase; AST, aspartate aminotransferase; TB, total bilirubin; DB, direct bilirubin; CRP, C-reactive protein; IL-6, interleukin-6; SAA, serum amyloid A; PCT, procalcitonin.

## 4 Discussion

Age has been proved to play an important role in the changes of inflammation and immune system (20, 21). The frequent demarcation to divide older or younger subjects in COVID-19 studies was age 60 or 65. The proportion of patients admitted to hospital, patients on oxygen, patients with severe diseases, and death rate in the population older than 60 years were significantly higher than that of population younger than 60 years during the four pandemic waves (14). However, the distinct responses to Omicron infection the population over 60 years are still lacking without comprehensive elucidation. In this retrospective observation study, we took the lead to investigate the various immunological and inflammatory responses to Omicron infection among different age populations. We observed that WBC and neutrophiles counts were elevated in Omicron infected patients compared to uninfected populations, while lymphopenia was observed after infection which was consistent with previous studies (1, 6). Notably, this phenomenon was more pronounced in the population older than 80 years old. Upon admission, pro-inflammatory cytokines, including CRP, IL-6, SAA, and PCT, were significantly increased in the Elder group when compared to the Adult and Old groups. During hospitalization, WBC continued to rise in the Old and Elder groups, which reflected a greater propensity for inflammation exacerbation among the elderly population in response to Omicron infection. In constract to the heightened inflammation response in the Elder group, the change of the cellular immunological response in the Old group exhibit an opposite pattern. The Old group demonstrated higher cell counts or proportions of T cell, T cell subpopulation, and B cell compared to the Elder group, suggesting that the cellular immunological response capacity might be somewhat suppressed in the Old population. However, the NK cell in the Elder group was higher compared to the Old group. During the first Asp614Gly infection wave, the effects of aging on the inflammation and immune response were manifested with elevated level of pro-inflammatory cytokines and depressed immune activation, which was quite similar to our findings (8, 22). In the aspect of humoral immunological response, only one marker, C3, showed a difference between the Old and Elder groups at Day 7. Jentsch-Ullrich et al. reported that the mean numbers of CD3^+^CD8^+^ T cells and CD19^+^ B cells declined significantly beyond an age of 50 years, whereas the mean counts of NK cells and CD4^+^/CD8^+^ ratio increased significantly beyond the age of 50 (23). Compared with non-Asian population, the Asian population had a lower mean percentage of CD3 and CD4 cells, a lower CD4/CD8 ratio, and lower absolute CD4 lymphocytes (24). However, the references of these cellular immune markers in the age of 60 to 79 years and older than 80 years have not been investigated. Hence, further studies are needed to determine that to what extent the changes in cellular immunity for the Old and Elder groups are caused by Omicron infection.

We further analyzed the underlying correlation between the levels of these biomarkers and the Omicron infected/convalescent patients in different population. The Omicron positive patients in the Adult group had similar levels of biomarkers routinely used in clinic as that of the negative patients. One possible reason was that the immune and inflammation regulation system was finely tuned to the Omicron infection. As consequence, the recovery period for Omicron infection was shorter for the Adult group. For the Old group, Omicron negative patients had higher proportion of CD19^+^ B cells, higher levels of C3 and IL-17 compared with that of Omicron positive patients. On the contrary, the WBC counts, the proportion of CD3^+^CD8^+^ T cells, the levels of SAA and IgA, were lower for the Omicron negative patients in the Elder group compered with that of positive patients, except for the ratio of CD4^+^/CD8^+^, which was higher in the negative patients. These different changes in the level of biomarkers for the Old and Elder groups indicated that the immune and inflammation regulation was in disorder resulted by Omicron infection, which was not restored to the status before infection even in the convalescent stage.

The death rate of Omicron infection decreased compared with previous Asp614Gly, Beta, and Delta pandemic waves (14). We found that the overall mortality of Omicron infection patients was 5.0%, which was consistent with the death rate of 4.5% reported in one previous study (25). Jassat W et al. reported that the death rate of Omicron infection in South Africa was 10.7% (14), while the study conducted by Maslo C et al. reported the death rate was 2.7% (26). One possible reason for the different death rate might be that the patients were enrolled in different periods of Omicron wave. According to previous studies, every 10-years increase in age was associated with higher death risk (8, 27). This was also confirmed in our study that the Elder group has the highest death rate of 11.01% compared with that of the Adult and Old groups in our study. After analyzing the correlation of inflammatory and immune markers with clinical outcomes, factors that contribute to mortality were age, pro-inflammatory markers, and organ dysfunction markers. Conversely, higher lymphocyte counts, higher level of hemoglobin, total protein, and albumin might be helpful to reduce mortality. In summary, the Elder population infected with Omicron showed increased inflammatory and depressed immune response, which needs more clinical attention considering the high mortality.

Aiming to find clues to limit or prevent the transmission and severe COVID-19, many scientists and clinicians have made great efforts to elucidate the clinical and immune characteristics against SARS-CoV-2 infection. One research line was the immune response to SARS-CoV-2 vaccines in uninfected individuals (3, 18, 28, 29). SARS-CoV-2–specific CD4 ^+^ and CD8^+^ T cells responses were observed in vaccine recipients which probably contribute to protection against severe SARS-CoV-2 infection, whereas the subjects enrolled in these studies were 22 to 67 years old (3, 18, 28, 29). According to the severity and stages of Omicron infection, patients could be classified as asymptomatic, mild, severe infection, and convalescent stage. Asymptomatic patients showed reduced inflammatory response characterized with low circulating concentrations of cytokines as compared to symptomatic individuals (30). Peng et al. found that the mild infection cases had lower breadth and magnitude of T cell responses as compared with that of severe cases, which might have the protective immunity (10). Sekine et al. systematically mapped the different functional and phenotypic landscape of SARS-CoV-2-specific T cell responses in unexposed individuals, exposed family members, and patients with acute or convalescent COVID-19, suggesting that natural exposure or infection might prevent recurrent episodes of severe COVID-19 (11). Collectively, finely tuned inflammatory and immune regulation might have the potential to shorten the time taken for Omicron-infected patients turning negative. Furthermore, the different inflammatory and immune response to Omicron infection is valuable to guide the precise treatment for different age population.

This study have some limitations. First, the severity of Omicron infection was not classified in different age population, where more detailed classification of Omicron infection can provide more valuable information. Secondly, immunomodulatory therapy might have effect on the immune response. Thirdly, the correlation between average hospitalization and levels of biomarkers in Omicron positive and negative patients was not analyzed. Many factors contribute to the average hospitalization length for the population over 60 years of age, especially the comorbidities that could be exacerbated with the Omicron infection can prolong the hospitalization length. Finally, we did not collect the chronic conditions or comorbidities of the enrolled patients. However, the main purpose of our paper was to investigate the inflammatory and immune responses with age during hospitalization in Omicron infected patients. Previous study have proved that age is a strong factor for COVID-19 hospital admission and mortality and Elixhauser comorbidity index was not associated with severe disease in patients older than 65 years (8).

Overall, we conducted a comprehensive analysis to characterize the distinct profiles of inflammatory and immune responses to Omicron infection, which were found to vary significantly with age. The population over 80 years had a stronger inflammatory response but a weaker immune response, which might contribute to the high mortality. Moreover, our research highlight the diverse correlations between the levels of various biomarkers and Omicron infected/convalescent patients. Therefore, a comprehensively understanding of the various inflammatory and immune responses to Omicron infection based on age proposes the hope to develop distinct treatments tailored to different age groups.

## Data availability statement

The raw data supporting the conclusions of this article will be made available by the authors, without undue reservation.

## Ethics statement

The studies involving human participants were reviewed and approved by Ruijin Hospital Luwan Branch Ethics Committee of Shanghai JiaoTong University Schoo of Medicine. Written informed consent for participation was not required for this study in accordance with the national legislation and the institutional requirements.

## Author contributions

LZ, ZQ, and YL contributed to study design. CL, CHL WL, and XM contributed to literature search and collected and curated data. ZW, FL, JZ, and XQ contributed to data analysis and creation of tables and figures. LZ, ZQ, and YL verified the underlying data. LZ, CL, CHL, WL, XM, ZQ, and YL contributed to data interpretation. LZ and ZW drafted the initial manuscript and all other co-authors contributed scientific inputs equally towards the interpretation of the findings and the final draft of the manuscript. The corresponding author had full access to all the data in the study and had final responsibility for the decision to submit for publication. All authors contributed to the article and approved the submitted version.
