# Supplementary material for: Characterizing distinct profiles of immune and inflammatory response with age to Omicron infection
Source: Front Immunol. 2023 Jun 30;14:1189482. doi: 10.3389/fimmu.2023.1189482 (PMC10348361; doi:10.3389/fimmu.2023.1189482)
Supplement: Supplementary file 1 [file DataSheet_1.docx]

Supplementary Material

Characterizing Distinct Profiles of Immune and Inflammatory Response with Age to Omicron Infection

Lina Zhang^1,2,3†^, Zhanwen Wang^1,2,3†^, Feng Lyu^4^, Chun Liu^5^, Chunhui Li^2,6^, Wei Liu^1,2,3^, Xinhua Ma^1,2,3^, Jieyu Zhou^4^, Xinyu Qian^4^, Zhaoxin Qian^1,2,3‡^, Yong Lu^7‡^

^†^ **Correspondence:** Corresponding Author: Zhaoxin Qian，[xyqzx@csu.edu.cn](mailto:xyqzx@csu.edu.cn); Yong Lu, [18917762053@163.com](mailto:18917762053@163.com)

# Supplementary Figures and Tables

## Supplementary Figures

| 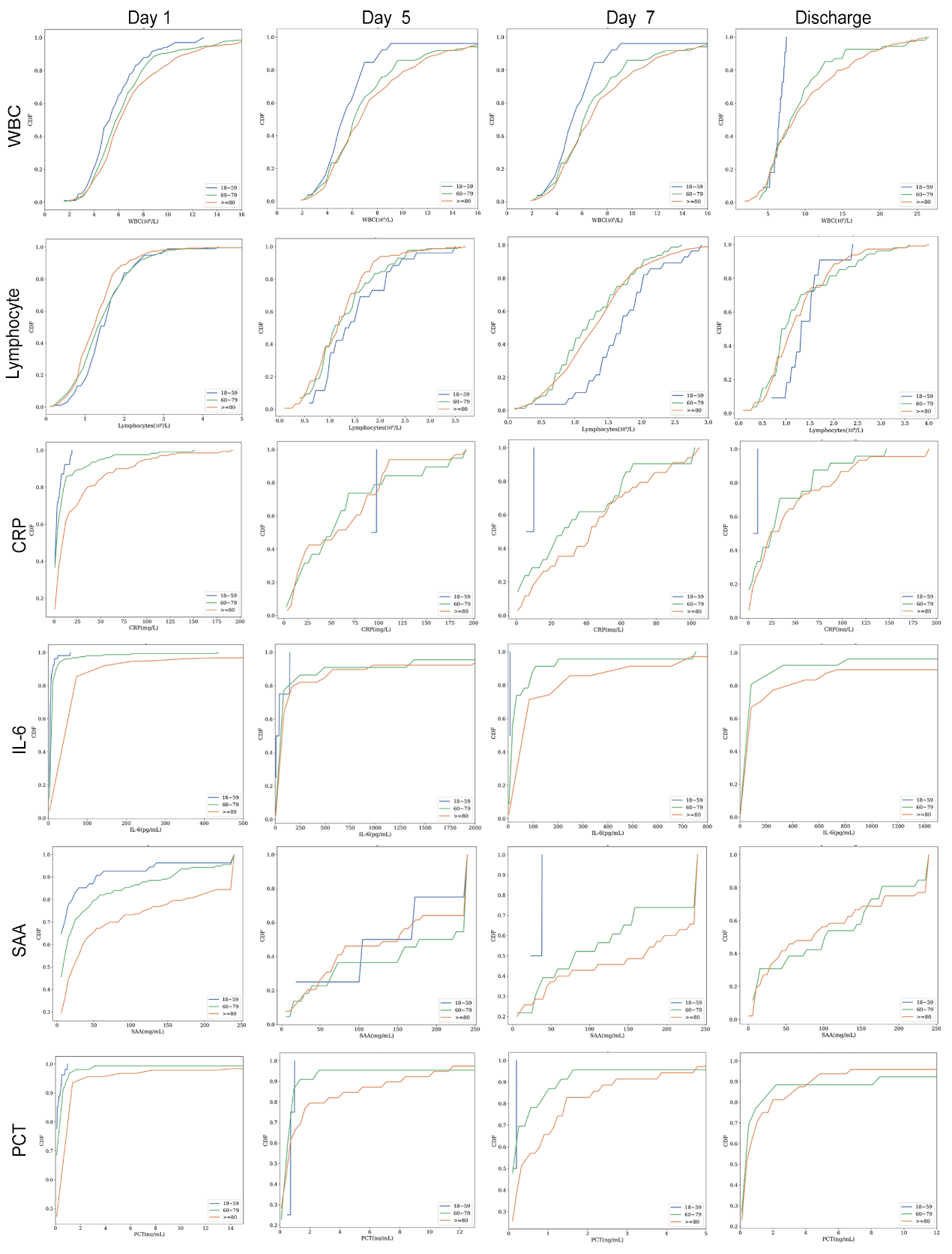 |
| --- |
| **Supplementary Figure 1.** The curves of cumulative distribution function (CDF) for WBC counts, lymphocyte counts, levels of CRP, IL-6, SAA, and PCT at Day 1, Day 5, and Day 7 after hospitalization as well as discharge in different age groups. The CDF curve of WBC for the Adult group was located on the upper left to the curves for Old and Elder groups at each timepoints. On the contrary, the CDF curve of lymphocyte for the Adult group was located on the lower right to the curves for Old and Elder groups at each timepoints. As the inflammatory cytokines were not regularly measured for the Adult group, the CDF curves for the Adult group were not as smooth as the Old and Elder groups. Bule line for Adult group, green line for Old group, and Orange line for Elder group. WBC = white blood cell; CRP = C-reactive protein; IL-6 = interleukin-6; SAA = serum amyloid A; PCT = procalcitonin. |

## Supplementary Tables

**Supplementary Table 1.** **Demographic characteristics and laboratory findings of Omicron infection patients at study entry.**

|  | Adult group  (n = 382) | | Old group  (n = 445) | | Elder group  (n = 472) | |
| --- | --- | --- | --- | --- | --- | --- |
|  | Mean (SD) | Median | Mean (SD) | Median | Mean (SD) | Median |
| Age, yr | 44.14 (10.26) | 45.0 | 69.98 (5.34) | 70.0 | 89.35 (4.93) | 89.0 |
| Sex, M/F | 132/250 | | 232/213 | | 167/305 | |
| Days taken for Ct value > 35, day | 4.91 (3.68) | 4.0 | 6.18 (4.63) | 5.0 | 6.80 (5.71) | 5.0 |
| White blood cell, 10^9^/L | 5.67 (2.21) | 5.28 | 6.53 (3.30) | 5.77 | 7.09 (3.72) | 6.1 |
| Neutrophils, 10^9^/L | 3.56 (2.06) | 3.1 | 4.44 (3.07) | 3.7 | 5.13 (3.56) | 4.1 |
| Lymphocytes, 10^9^/L | 1.52 (0.65) | 1.4 | 1.43 (0.68) | 1.4 | 1.30 (0.65) | 1.2 |
| Platelets, 10^9^/L | 217.60 (70.86) | 217.0 | 203.36 (80.81) | 198.0 | 201.79 (83.00) | 188.5 |
| Hb, g/L | 132.1 (20.16) | 135.0 | 126.8 (19.85) | 130.0 | 116.2 (19.31) | 118.5 |
| TP, g/L | 70.35 (6.70) | 70.00 | 67.62 (6.92) | 68.00 | 65.27 (7.85) | 66.00 |
| Albumin, g/L | 43.39 (4.41) | 43.0 | 39.88 (5.99) | 41.0 | 36.17 (5.75) | 36.0 |
| ALT, U/L | 27.49 (21.73) | 21.0 | 23.67 (17.63) | 19.0 | 27.38 (67.50) | 16.0 |
| AST, U/L | 24.49 (12.76) | 22.0 | 25.51 (18.06) | 22.0 | 30.74 (47.50) | 22.0 |
| Total bilirubin, µmol/L | 11.48 (6.73) | 10.0 | 12.94 (12.34) | 10.0 | 13.73 (11.26) | 11.0 |
| Direct bilirubin, µmol/L | 1.84 (3.11) | 0.70 | 3.87 (9.28) | 1.9 | 4.58 (7.70) | 2.75 |
| γ-GT, U/L | 30.14 (22.79) | 23.0 | 43.03 (103.6) | 23.0 | 34.13 (48.20) | 20.0 |
| Serum creatinine, µmol/L | 185.0 (428.9 ) | 62.00 | 88.47 (151.1) | 62.50 | 101.3 (141.6) | 72.50 |
| Blood urea nitrogen, mmol/L | 6.94 (8.80) | 4.5 | 6.97 (7.42) | 5.25 | 9.60 (8.70) | 7.1 |
| CRP, mg/L | 3.84 (4.92) | 1.47 | 10.14 (21.81) | 1.92 | 23.57 (35.11) | 9.00 |
| IL-6, pg/mL | 6.52 (7.97) | 4.30 | 13.42 (40.83) | 4.90 | 66.03 (284.7) | 7.40 |
| SAA, mg/mL | 24.47 (49.73) | 6.00 | 39.66 (63.28) | 7.34 | 70.38 (87.58) | 21.34 |
| PCT, ng/mL | 0.16 (0.17) | 0.10 | 0.39 (2.03) | 0.10 | 1.15 (5.82) | 0.11 |

Definition of abbreviations: Hb = hemoglobin; TP = total protein; ALT = Alanine aminotransferase; AST = Aspartate aminotransferase; γ-GT = γ-Glutamyltransferase; CRP = C-reactive protein; IL-6 = interleukin-6; SAA = serum amyloid A; PCT = procalcitonin.

**Supplementary Table 2. Demographic characteristics and laboratory findings of the uninfected control population.**

|  | Adult group  (n = 2094) | | Old group  (n = 2097) | | Elder group  (n = 2682) | |
| --- | --- | --- | --- | --- | --- | --- |
|  | Mean (SD) | Median | Mean (SD) | Median | Mean (SD) | Median |
| Age, yr | 44.0 (9.74) | 45.0 | 67.75 (5.25) | 67.0 | 85.33 (5.27) | 84.0 |
| Sex, M/F | 1106 / 988 | | 1220 / 877 | | 1739 / 942 | |
| White cell count, 10^9^/L | 6.15 (1.67) | 5.90 | 6.12 (1.63) | 5.9 | 6.28 (1.68) | 6.1 |
| Neutrophils, 10^9^/L | 3.58 (1.29) | 3.4 | 3.59 (1.32) | 3.4 | 3.67 (1.31) | 3.5 |
| Lymphocytes, 10^9^/L | 1.99 (0.58) | 1.9 | 1.92 (0.6) | 1.9 | 1.94 (0.64) | 1.8 |
| Platelets, 10^9^/L | 215.02 (56.27) | 210.0 | 198.67 (66.11) | 193.0 | 176.92 (56.42) | 172.0 |
| Hb, g/L | 142.04 (16.65) | 142.0 | 138.54 (14.27) | 138.0 | 134.69 (13.81) | 134 |
| TP, g/L | 74.65 (4.05) | 74.60 | 73.50 (4.38) | 73.5 | 72.98 (4.40) | 73.70 |
| Albumin, g/L | 46.6 (3.04) | 46.6 | 44.59 (3.14) | 44.8 | 43.56 (3.2) | 43.7 |
| ALT, U/L | 25.86 (20.48) | 20.0 | 24.5 (15.32) | 20.4 | 20.77 (15.94) | 17.4 |
| AST, U/L | 24.73 (11.66) | 22.3 | 27.38 (12.28) | 24.4 | 25.77 (11.23) | 24.0 |
| Total bilirubin, µmol/L | 12.08 (5.67) | 11.15 | 12.42 (6.34) | 11.1 | 12.8 (5.32) | 11.8 |
| Serum creatinine, µmol/L | 79.96 (26.49 ) | 78.05 | 81.78 (30.49) | 79 | 91.64 (29.74) | 87.8 |
| Blood urea nitrogen, mmol/L | 4.7 (1.51) | 4.57 | 5.36 (1.97) | 5.14 | 5.77 (1.99) | 5.5 |
| C-reactive protein, mg/L | 3.12 (3.05) | 2.11 | 5.22 (14.69) | 2.34 | 7.48 (12.88) | 3.5 |

Definition of abbreviations: Hb = hemoglobin; TP = total protein; ALT = Alanine aminotransferase; AST = Aspartate aminotransferase.

**Supplementary Table 3. The characteristic change for white blood cell, lymphocyte, and inflammatory cytokines in different age during hospitalization.**

|  |  | WBC, 10^9^/L | Lympho-cyte, 10^9^/L | CRP, mg/L | IL-6, pg/mL | SAA, mg/mL | PCT, ng/mL |
| --- | --- | --- | --- | --- | --- | --- | --- |
|  |  | Mean (SD) Median | Mean (SD) Median | Mean (SD) Median | Mean (SD) Median | Mean (SD) Median | Mean (SD) Median |
| Day 1 | Adult group | 5.67 (2.21) 5.28 | 1.52  (0.64)  1.40 | 3.84 (4.92) 1.47 | 6.52  (7.97)  4.3 | 24.47 (49.73) 6.0 | 0.16 (0.17) 0.1 |
|  | Old group | 6.53 (3.30) 5.77 | 1.43  (0.68)  1.40 | 10.14 (21.81) 1.92 | 13.42 (40.83)  4.9 | 39.66 (63.28) 7.34 | 0.39 (2.03) 0.1 |
|  | Elder group | 7.09 (3.72) 6.1 | 1.30  (0.65)  1.2 | 23.57 (35.11) 9.00 | 66.03 (284.7)  7.4 | 70.38 (87.58) 21.34 | 1.15 (5.8) 0.11 |
| Day 5 | Adult group | 5.91 (3.16) 5.25 | 1.5  (0.70) 1.35 | / | 49.03 (66.31) 22.45 | 171.3 (68.42) 170.8 | 0.74 (0.20)  0.73 |
|  | Old group | 7.27 (4.11) 6.18 | 1.29  (0.65)  1.1 | 62.20 (56.84)49.00 | 296.49 (878.1) 15.55 | 154.37 (96.18) 199.76 | 1.39 (4.35) 0.25 |
|  | Elder group | 7.61 (3.92) 6.52 | 1.21  0.6  1.2 | 60.02 (49.77) 55.90 | 359.37 (930.0) 51.9 | 134.36 (92.37) 151.27 | 2.08 (3.62) 0.53 |
| Day 7 | Adult group | 6.0 (1.42)  6.0 | 1.7  (0.55)  1.7 | / | / | / | / |
|  | Old group | 7.39 (3.45)  6.68 | 1.25  (0.56)  1.2 | 35.03 (31.55) 26.79 | 64.6 (157.0) 16.0 | 108.58 (93.08) 80.0 | 0.66 (1.58)  0.11 |
|  | Elder group | 7.75 (4.13) 6.76 | 1.37  (1.03)  1.3 | 43.85 (31.55) 42.11 | 221.2 (682.5) 32.8 | 133.25 (101.4) 169.59 | 1.0 (1.37)  0.32 |
| Discharge | Adult group | 6.36 (0.92) 6.45 | 1.39  (0.44)  1.3 | / | / | / | / |
|  | Old group | 9.34 (5.06) 7.99 | 1.27  (0.79) 0.95 | 35.55 (38.70) 24.85 | 220.34 (786.4) 10.75 | 109.61 (86.86) 102.0 | 2.13 (5.23)  0.22 |
|  | Elder group | 10.07 (5.60) 8.42 | 1.25  (0.7)  1.1 | 45.6 (48.51) 22.0 | 444.51 (1046.22 )  19.9 | 106.1 (92.10) 86.04 | 1.64 (3.22) 0.38 |

Definition of abbreviations: WBC = White blood cell; CRP = C-reactive protein; IL-6 = interleukin-6; SAA = serum amyloid A; PCT = procalcitonin.

**Supplementary Table 4. The characteristic change for the cellular and humoral immune response and inflammatory cytokines in different age during hospitalization.**

|  | Day 3 | | Day 7 | | Discharge | |
| --- | --- | --- | --- | --- | --- | --- |
|  | Old group | Elder group | Old group | Elder group | Old group | Elder group |
| CD3^+^ T, cells/µL | 1075.0 (506.1), 921.5 | 830.0 (301.0), 866.5 | 1040.62 (424.3), 976.0 | 720.52 (309.6), 707.0 | 893.21 (428.3), 963.0 | 868.22 (359.0), 875.0 |
| CD3^+^ T proportion | 66.8 (11.25), 68.9 | 64.63 (11.28), 65.95 | 72.4 (9.68), 71.7 | 66.23 (9.07), 66.4 | 68.82 (12.25), 64.5 | 68.74 (9.15), 69.3 |
| CD4^+^ T, cells/µL | 628.8 (329.0), 590.0 | 499.5 (161.3), 482.0 | 628.6 (274.7) , 615.5 | 432.7 (182.3), 422.0 | 573.3 (270.3), 574.0 | 528.4 (236.2), 474.0 |
| CD3^+^CD4^+^ T proportion | 38.24 (9.38), 38.05 | 40.19 (10.41), 37.5 | 43.33 (8.18), 42.75 | 40.17 (9.89), 40.6 | 44.14 (11.27), 40.5 | 42.93 (12.27), 44.0 |
| CD8^+^ T, cells/µL | 414.1 (186.1), 382.0 | 329.5 (215.8), 279.5 | 393.3 (181.6), 370.0 | 291.7 (206.5), 253.0 | 323.5 (215.9), 271.0 | 343.4 (231.8), 314.0 |
| CD3+ CD8+ T proportion | 27.1 (8.40), 26.3 | 24.29 (10.44), 22.0 | 27.98 (9.26), 26.45 | 25.41 (9.49), 23.9 | 24.79 (10.27), 22.8 | 26.13 (10.78), 22.5 |
| CD4+/CD8+ | 1.58 (0.69), 1.50 | 2.04 (1.16), 1.82 | 1.73 (0.68), 1.72 | 1.86 (0.94), 1.76 | 2.14 (1.12), 1.96 | 1.99 (1.08), 1.82 |
| CD19^+^ B, cells/µL | 168.0 (101.6), 156.5 | 113.4 (97.09), 87.5 | 164.1 (127.1), 117.0 | 105.1 (75.62), 90.0 | 146.1 (118.7), 127.0 | 113.3 (56.76), 97.0 |
| CD19^+^B proportion | 11.32 (5.22), 10.55 | 9.12 (5.27), 7.75 | 11.50 (6.58), 10.4 | 9.59 (5.99), 7.9 | 11.46 (7.43), 11.3 | 9.49 (4.67), 8.2 |
| NK, cells/µL | 279.1 (154.7), 268.5 | 294.5 (170.9), 259.0 | 200.6 (111.5), 210.5 | 263.2 (160.4), 222.0 | 243.1 (201.0), 185.0 | 275.3 (172.7), 257.0 |
| NK proportion | 19.44 (9.11), 18.10 | 23.85 (10.97), 23.1 | 14.98 (8.24), 14.85 | 23.14 (7.73), 22.50 | 18.39 (10.41), 16.50 | 20.97 (9.67), 21.00 |
| C3, g/L | 1.08 (0.27), 1.08 | 1.00 (0.20), 0.99 | 1.93 (0.25). 1.22 | 0.94 (0.20), 0.93 | 1.31 (0.33), 1.16 | 0.98 (0.21), 0.98 |
| C4, g/L | 0.26 (0.09), 0.27 | 0.28 (0.10), 0.26 | 0.28 (0.09), 0.28 | 0.25 (0.08), 0.25 | 0.28 (0.10), 0.28 | 0.26 (0.11), 0.24 |
| IgA, g/L | 2.50 (1.23), 2.40 | 2.72 (0.95), 2.60 | 2.98 (1.95), 2.48 | 2.64 (1.15), 2.64 | 2.87 (1.18), 2.56 | 3.16 (1.51), 2.83 |
| IgM, g/L | 0.83 (0.69), 0.67 | 0.86 (0.60), 0.68 | 0.85 (0.71), 0.69 | 0.87 (0.64), 0.69 | 0.71 (0.37), 0.63 | 0.78 (0.42), 0.64 |
| IgG, g/L | 11.69 (3.94), 11.60 | 12.24 (3.32), 12.25 | 12.66 (3.64), 12.50 | 11.85 (3.69), 11.60 | 11.79 (3.92), 11.25 | 13.04 (3.86), 13.20 |

Definition of abbreviations: NK = nature kill cell; C3 = Complement 3; C4 = Complement 4.

**Supplementary Table 5. The correlation between Omicron infection stage and immunological and inflammatory cytokines in different age population**

|  |  | Adult group | Old group | Elder group |
| --- | --- | --- | --- | --- |
|  |  | Mean (SD)  Median | Mean (SD)  Median | Mean (SD)  Median |
| WBC, 10^9^/L | Negative | 6.32 (2.69)  5.4 | 6.48 (2.35)  6.35 | 6.91 (4.01)  5.91 |
|  | Positive | 5.77(2.14)  5.48 | 7.47(3.98)  6.38 | 8.16(4.61)  6.79 |
| Lymphocyte, 10^9^/L | Negative | 1.48 (0.43) 1.60 | 1.33 (0.42) 1.20 | 1.20 (0.54) 1.05 |
|  | Positive | 1.53 (0.63) 1.5 | 1.35 (0.96) 1.20 | 1.24 (0.77) 1.20 |
| CRP, mg/L | Negative | 8.25 (6.92) 8.47 | 10.99 (9.77) 9.20 | 21.11 (25.20) 11.42 |
|  | Positive | 7.89 (19.69) 1.99 | 25.37 (36.40) 8.00 | 34.40 (38.58) 19.55 |
| IL-6, pg/L | Negative | 4.78 (1.93) 4.10 | 17.89 (16.83) 11.90 | 26.10 (43.11) 8.90 |
|  | Positive | 9.30 (19.55) 4.75 | 57.12 (280.80) 6.50 | 157.20 (555.60) 12.40 |
| SAA, mg/L | Negative | 22.45 (22.16) 15.45 | 53.67 (83.35) 21.00 | 53.59 (67.05) 6.65 |
|  | Positive | 30.54 (57.68) 6.00 | 76.42 (87.30) 26.62 | 93.97 (92.27) 48.47 |
| PCT, ng/L | Negative | 0.16 (0.11) 0.10 | 0.36 (0.33) 0.20 | 0.90 (2.33) 0.10 |
|  | Positive | 0.21 (0.24) 0.10 | 1.14 (4.85) 0.10 | 1.41 (4.76) 0.22 |
| CD3^+^CD8^+^, cells/µL | Negative | / | 22.63 (5.03) 21.75 | 13.93 (7.60) 14.20 |
|  | Positive | / | 25.46 (9.24) 23.55 | 26.38 (9.59) 26.00 |
| CD4^+^/CD8^+^ | Negative | / | 1.88 (0.91) 1.63 | 3.79 (1.68) 3.51 |
|  | Positive | / | 2.02 (0.98) 1.95 | 1.83 (0.88) 1.60 |
| CD19^+^, cells/µL | Negative | / | 15.62 (6.23) 13.15 | 8.13 (2.29) 9.20 |
|  | Positive | / | 10.79 (5.77) 10.55 | 9.65 (6.04) 7.90 |
| C3, g/L | Negative | / | 1.35 (0.26) 1.28 | 0.90 (0.08) 0.96 |
|  | Positive | / | 1.10 (0.24) 1.14 | 0.96 (0.19) 0.96 |
| IgA, g/L | Negative | / | 2.89 (1.19) 3.11 | 1.65 (0.71) 1.33 |
|  | Positive | / | 2.64 (1.40) 2.45 | 2.94 (1.34) 2.78 |
| IL-17, pg/mL | Negative | / | 20.57 (4.22) 21.95 | 14.23 (1.75) 14.70 |
|  | Positive | / | 14.58 (4.82) 13.70 | 17.34 (9.17) 15.10 |

Definition of abbreviations: WBC = white blood cell; CRP = C-reactive protein; IL-6 = interleukin-6; SAA = serum amyloid A; PCT = procalcitonin; SAA = serum amyloid A; C3 = complement 3; IL-17 = interleukin 17.
